# Notes and Notices

**Published:** 1891-09

**Authors:** 


### NOTES AND NOTICES.

FOR SALE.—Volume Tenth (1890) of THE HOMŒOPATHIC PHYSICIAN complete, unbound. Also Volume Ninth (1889), wanting April, October, and November numbers, and Volume Eleventh (1891), January to July. These all belong to the estate of the late Dr. Wm. A. Hawley, of Syracuse, New York. Address M. E. H., care of THE HOMŒOPATHIC PHYSICIAN, 1125 Spruce Street, Philadelphia, Pa.

THE AMERICAN PUBLIC HEALTH ASSOCIATION will hold its nineteenth annual meeting at Kansas City, Missouri, Kansas, October 20th, 21st, 22d, 23d, 1891. The Executive Committee have selected the following topics for consideration at said meeting: 1. Sanitary Construction in House Architecture. (a) Heating. (b) Lighting. (c) Drainage. (d) Ventilation. 2. Railroad Sanitation. 3. Meat Supplies. 4. Milk Supplies of Cities. 5. Arsenical Papers and Fabrics. 6. Isolation Hospitals for Infectious Diseases in Cities. 7. Papers upon any of the subjects upon which special committees have been appointed. 8. Papers on Miscellaneous Sanitary and Hygienic Subjects. All papers will be received by the Executive Committee subject to the requirements of the By-Laws. Preference will be given, however, to papers upon the subjects selected by the Committee in making up the daily programme of the meeting. All persons who propose to present papers at the next meeting of the Association will be governed by the following By-Laws of the Executive Committee: "4. All papers presented to the Association must be either printed, type-written, or in plain handwriting, and be in the hands of the Secretary at least twenty days prior to the annual meeting, to insure their critical examination as to their fulfilling the requirements of the Association." All communications relating to local matters should be sent to E. R. Lewis, M. D., Chairman Local Committee of Arrangements, Kansas City, Mo. Blank applications for membership may be obtained from the Secretary, Irving A. Watson, M. D., Concord, N. H.

THE MICHIGAN STATE HOMŒOPATHIC MEDICAL SOCIETY has elected the following officers: President, J. C. Wood, of Ann Arbor; Vice-President, H. C. Brigham, of Grand Rapids, and A. B. Cornell, of Kalamazoo; General Secretary, Harold Wilson, of Detroit; Corresponding Secretary, W. A. Ploglase, of Detroit; Treasurer, H. M. Warren, of Jonesville; member of the Board of Control, W. M. Bailey, of Detroit.

THE GRAND RAPIDS, MICH., COLLEGE OF HOMŒOPATHIC PHYSICIANS AND SURGEONS has elected the following officers: President, Dr. H. C. Brigham; First Vice-President, Dr. I. J. Whitfield; Second Vice-President, Dr. Francis S. Hillyer; Secretary and Treasurer, Dr. F. L. Hoag. Various subjects of interest to the doctors were discussed.

